# Gut microbiota-derived metabolites in keloid and hypertrophic scarring

**DOI:** 10.3389/fmicb.2025.1644758

**Published:** 2025-09-05

**Authors:** Junjie Jin, Zhenlong Zheng

**Affiliations:** Department of Dermatology, Yanbian University Hospital, Jilin, China

**Keywords:** skin–gut axis, pathological scarring, gut microbiome, immunomodulation, microbiome therapies

## Abstract

Keloids and hypertrophic scars are fibro-proliferative skin disorders that arise from aberrant wound healing and are characterized by excessive collagen deposition and chronic inflammation. Although traditionally viewed as strictly local cutaneous phenomena, growing evidence suggests that systemic influences—particularly the gut microbiota and its metabolites—may influence scar pathogenesis. The gut microbiota produces a wide range of bioactive compounds, including short-chain fatty acids (SCFAs), bile acids, and tryptophan derivatives, which are hypothesized to modulate immune responses and pro-fibrotic signaling pathways such as TGF-*β* and Wnt/β-catenin. Observations from systemic fibrotic disorders—for example, liver and pulmonary fibrosis—link microbial dysbiosis to aberrant extracellular-matrix remodeling. Although direct evidence in skin fibrosis is still limited, recent multi-omics analyses and microbiota-transplantation studies imply that gut-derived factors may influence dermal fibroblast behavior. This review therefore synthesizes the emerging conceptual and mechanistic connections between gut microbial metabolites and pathological scar formation, proposes a possible skin-gut-fibrosis axis, and outlines potential avenues for therapeutic intervention in keloids and hypertrophic scars.

## 1 Introduction

Pathological scarring, encompassing keloids and hypertrophic scars, arises from dysregulated wound healing and is characterized by excessive extracellular matrix accumulation and chronic inflammation. Clinically, hypertrophic scars remain confined to the original wound boundaries, presenting as firm, elevated lesions, while keloids extend beyond the wound edges, forming irregular plaques that may cause pain, pigmentation changes, or functional limitation (Gauglitz et al., 2011). These lesions often result in the loss of skin appendages such as hair follicles and sebaceous glands (Ogawa, 2017). Keloids are more commonly observed in individuals with darker skin tones, particularly among populations of African and African descent, with estimated prevalence ranging from 5 to 16% (Brown et al., 2008). Population-based studies have reported prevalence rates as high as 16% in African and Afro-Caribbean populations—for instance, up to 16% in the Democratic Republic of Congo (Kiprono et al., 2015), and approximately 6–10% among African Americans in the United States (Olopoenia et al., 2024). Beyond the visible impact, pathological scarring imposes significant psychological burdens, including anxiety, depression, and social withdrawal, thereby impairing overall quality of life (Kim et al., 2022).

Despite the availability of multiple treatment modalities—such as pressure therapy, silicone dressings, corticosteroid injections, laser therapy, radiotherapy, and surgical excision—their effectiveness is often limited by high recurrence rates and side effects. For instance, corticosteroids suppress fibroblast activity and inflammation but may induce skin atrophy or dyschromia, with a high rate of relapse upon cessation (Mustoe et al., 2002). Laser therapy and surgical removal offer cosmetic benefits but are frequently ineffective for established or extensive lesions unless combined with adjunctive therapies such as intralesional corticosteroid injections, postoperative radiotherapy, pressure therapy, or silicone gel sheeting, which aim to reduce recurrence and improve outcomes (Alster and Tanzi, 2003; Ogawa, 2010). These limitations motivate exploration of systemic drivers beyond local dermal factors in order to identify disease-modifying strategies.

Amid these therapeutic challenges, the human microbiome has emerged as a compelling framework for understanding and potentially modulating fibrotic diseases. The gut microbiota is a key regulator of host immunity, systemic inflammation, and tissue repair processes (Grice and Segre, 2011). Both the gut and skin function as immunologically active barrier organs, forming a bidirectional “skin-gut axis” that has been implicated in inflammatory skin conditions such as atopic dermatitis, acne, and psoriasis (O’Neill et al., 2016; Salem et al., 2018). Recent studies further suggest that microbial dysbiosis and altered metabolic outputs—such as short-chain fatty acids and bile acids—may influence dermal fibroblast behavior and contribute to fibrosis via immune polarization and activation of profibrotic signaling pathways (Poutahidis et al., 2013).

**Figure 1:**
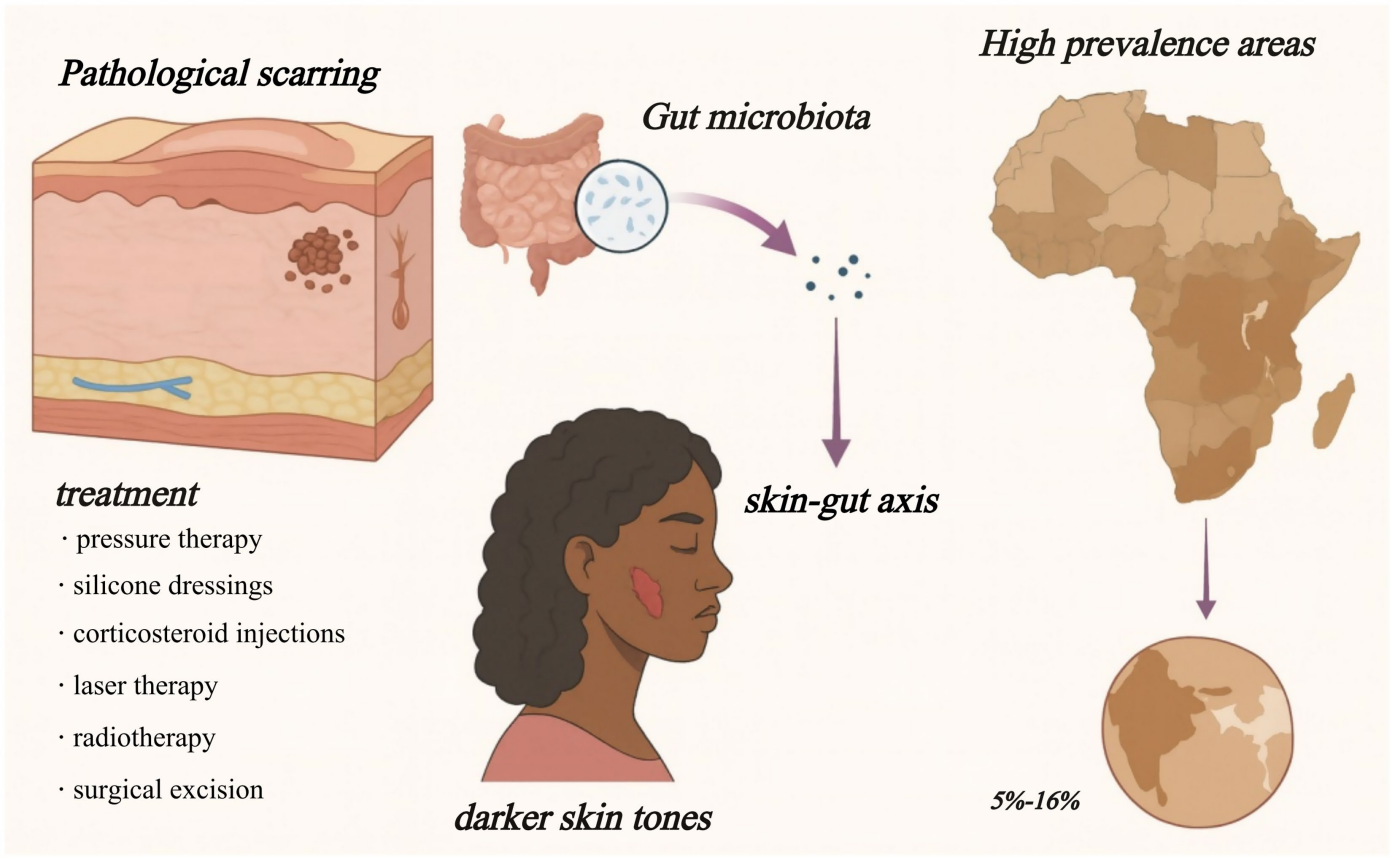
Skin–gut axis in pathological scarring. Keloids and hypertrophic scars are most common in darker-skinned populations (5–16% prevalence in parts of Africa) and respond poorly to current therapies. Growing evidence links gut-microbiota metabolites to scar-driving immune and fibroblast changes, suggesting a systemic target for future treatment.

Although direct mechanistic links between gut microbiota and skin fibrosis remain insufficiently explored, early multi-omics and transplantation studies indicate that gut-derived factors may modulate fibroblast activity and extracellular matrix remodeling. Clarifying these interactions may open new avenues for microbiome-based precision therapies targeting keloid and hypertrophic scar formation (Figure 1).

## 2 Local drivers of pathological scarring

The formation of pathological scars is a complex and multifactorial process, fundamentally driven by the overactivation and dysregulation of the wound healing response. Traditional research has primarily focused on the interplay among dysregulated cell signaling pathways, aberrant cellular phenotypes, immune microenvironment imbalances, and mechanical stressors (Ogawa, 2022). In recent years, emerging studies have highlighted the importance of epigenetic regulation and metabolic reprogramming in the pathogenesis of pathological scarring, offering new molecular insights and potential therapeutic targets.

### 2.1 Fibro-mechanical circuitry

Pathological scarring, including hypertrophic scars and keloids, is a prototypical fibro proliferative disorder resulting from chronic dysregulation of wound healing. Unlike the transient inflammation observed in physiological repair, pathological scars exhibit persistent immune infiltration—particularly of M2 macrophages, mast cells, and Th2 lymphocytes—which together sustain a pro-fibrotic microenvironment via cytokines such as IL-4, IL-13, and TNF-*α* (Willenborg and Eming, 2014).

Central to this process is fibroblast hyperactivity, characterized by enhanced proliferation, apoptosis resistance, and overproduction of extracellular matrix (ECM) components (Darby et al., 2014). The TGF-*β*/Smad pathway plays a pivotal role, maintaining myofibroblast activation and collagen deposition (Lee et al., 2020). Recent work has identified macrophage-to-myofibroblast transition (MMT), mediated by TGF-β/Smad3 signaling, as an additional source of contractile fibroblasts in cutaneous fibrosis (Jia et al., 2025).

Mechanical tension further exacerbates fibrosis. Forces generated in high-stress anatomical regions (e.g., chest, shoulders) activate latent TGF-β1 and drive sustained myofibroblast differentiation and contractile matrix remodeling (Walker et al., 2020). TGF-β1 also suppresses ECM degradation by inhibiting matrix metalloproteinases (MMPs) and upregulating tissue inhibitors of metalloproteinases (TIMPs), favoring matrix accumulation (Xue and Jackson, 2015). Mechanotransduction through integrin–FAK–YAP/TAZ signaling has been shown to reinforce this fibrotic loop, particularly under persistent strain (Pakshir et al., 2019; Harn et al., 2019).

Fibrogenic signaling is further amplified by downstream effectors such as connective tissue growth factor (CTGF) and platelet-derived growth factor (PDGF), the latter promoting fibroblast proliferation, chemotaxis, and ECM synthesis (Shi-Wen et al., 2008; Kohlhauser et al., 2024). Notably, single-cell RNA sequencing (scRNA-seq) studies have recently uncovered mechanosensitive fibroblast subpopulations enriched in hypertrophic scars and keloids, distinguished by elevated expression of ACTA2, YAP1, and fibrosis-associated collagen genes (Deng et al., 2025; Zhao et al., 2025). These populations exhibit heightened responsiveness to mechanical stress and may represent key therapeutic targets.

Finally, genetic predisposition plays a non-negligible role in scarring susceptibility. Polymorphisms in components of the TGF-*β* pathway are disproportionately reported among individuals of African, Asian, and Hispanic descent (Hu et al., 2018), offering an explanation for observed ethnic disparities in keloid incidence.

Despite these advances, several critical gaps remain. The dynamic transitions among fibroblast subtypes under mechanical strain are not fully understood, and the integrative roles of epigenetic regulation and biomechanical feedback loops remain incompletely mapped. Recent studies have also identified epigenetic mechanisms—such as DNA methylation, histone modification, and non-coding RNA regulation—as key contributors to persistent fibroblast activation. For instance, hypermethylation of antifibrotic genes (e.g., SMAD7) and upregulation of pro-fibrotic microRNAs such as miR-21 and miR-199a enhance TGF-*β* signaling and collagen production in keloid fibroblasts (Li et al., 2021). Specifically, miR-21 promotes keloid fibrosis by downregulating SMAD7, thereby enhancing TGF-β/Smad signaling (Wu et al., 2019), and miR-199a-5p is downregulated in keloid tissue, with its restoration shown to inhibit keloid fibroblast proliferation and alter the cell cycle. Additionally, metabolic reprogramming—including increased glycolysis and glutaminolysis—has been shown to support the bioenergetic and biosynthetic needs of activated fibroblasts in scar tissue, further sustaining their fibrogenic phenotype (Li et al., 2018).

### 2.2 Immune dysregulation

The development of pathological scars, including hypertrophic scars and keloids, is closely linked to a dysregulated immune microenvironment. Among the various immune components, abnormal macrophage polarization plays a central role. In normal skin wound healing, macrophages transition from the pro-inflammatory M1 phenotype in the early inflammatory phase to the anti-inflammatory M2 phenotype during tissue remodeling—a switch essential for effective repair and angiogenesis (Faulknor et al., 2017; Gao et al., 2023). This balance is disrupted in pathological scars, where persistent infiltration of M2 macrophages—particularly the M2a and M2c subsets marked by CD163 and CD206—is commonly observed. These M2 macrophages maintain a pro-fibrotic environment by secreting mediators such as TGF-*β*1 and PDGF, which promote fibroblast proliferation, myofibroblast differentiation, and ECM overproduction (Shi et al., 2024). Elevated Th2 cytokines (e.g., IL-4, IL-13), damage-associated molecular patterns (DAMPs), and altered ECM composition further drive this M2 bias (Chao et al., 2023).

Beyond innate immunity, adaptive immune dysregulation—particularly involving the Th17/Treg axis—is a hallmark of pathological scarring. Keloid tissues exhibit increased Th17 cell infiltration and activity, characterized by overexpression of IL-17, IL-6, and TNF-*α*, all of which contribute to fibroblast activation and collagen deposition. Conversely, Tregs, which normally exert antifibrotic functions via IL-10 and TGF-β, are numerically and functionally diminished, leading to a heightened Th17/Treg ratio (Bitterman et al., 2024; Deng et al., 2025). Emerging data indicate that IL-23 and IL-1β act upstream of Th17 polarization, amplifying IL-17A/F output and sustaining chronic inflammation (Dufour et al., 2020). This imbalance not only fuels fibrosis but also represents a promising therapeutic entry point.

The cytokine milieu is further skewed toward fibrosis. IL-6, in particular, is markedly upregulated in hypertrophic scars and correlates with symptom severity such as pruritus (Johnson et al., 2020). It drives fibroblast proliferation, collagen synthesis, and M2 polarization through the JAK/STAT3 axis and facilitates Th17 cell differentiation. In contrast, IL-10—an anti-fibrotic cytokine—tends to be deficient or functionally impaired in pathological scars (Shen et al., 2025). Notably, preclinical studies have demonstrated that local delivery of IL-10 can alleviate dermal fibrosis by modulating the IL-10R/STAT3 axis and suppressing TLR4/NF-κB signaling, suggesting therapeutic potential for pathological scars (Shi et al., 2021).

Persistent expression of upstream pro-inflammatory cytokines like TNF-*α* and IL-1β also contributes to chronic inflammation. IL-1β, in particular, is sustained through excessive NLRP3 inflammasome activation, promoting fibroblast activation and immune cell recruitment (Hameedi et al., 2025).

In summary, pathological scarring is driven by a chronically activated immune microenvironment characterized by M2 macrophage predominance, Th17/Treg imbalance, and a profibrotic cytokine milieu. Therapeutic modulation of these pathways—such as IL-6 or IL-17A inhibition, or restoration of IL-10 signaling and Treg function—holds promise for the treatment of refractory hypertrophic scars and keloids (Mu et al., 2024).

**Figure 2:**
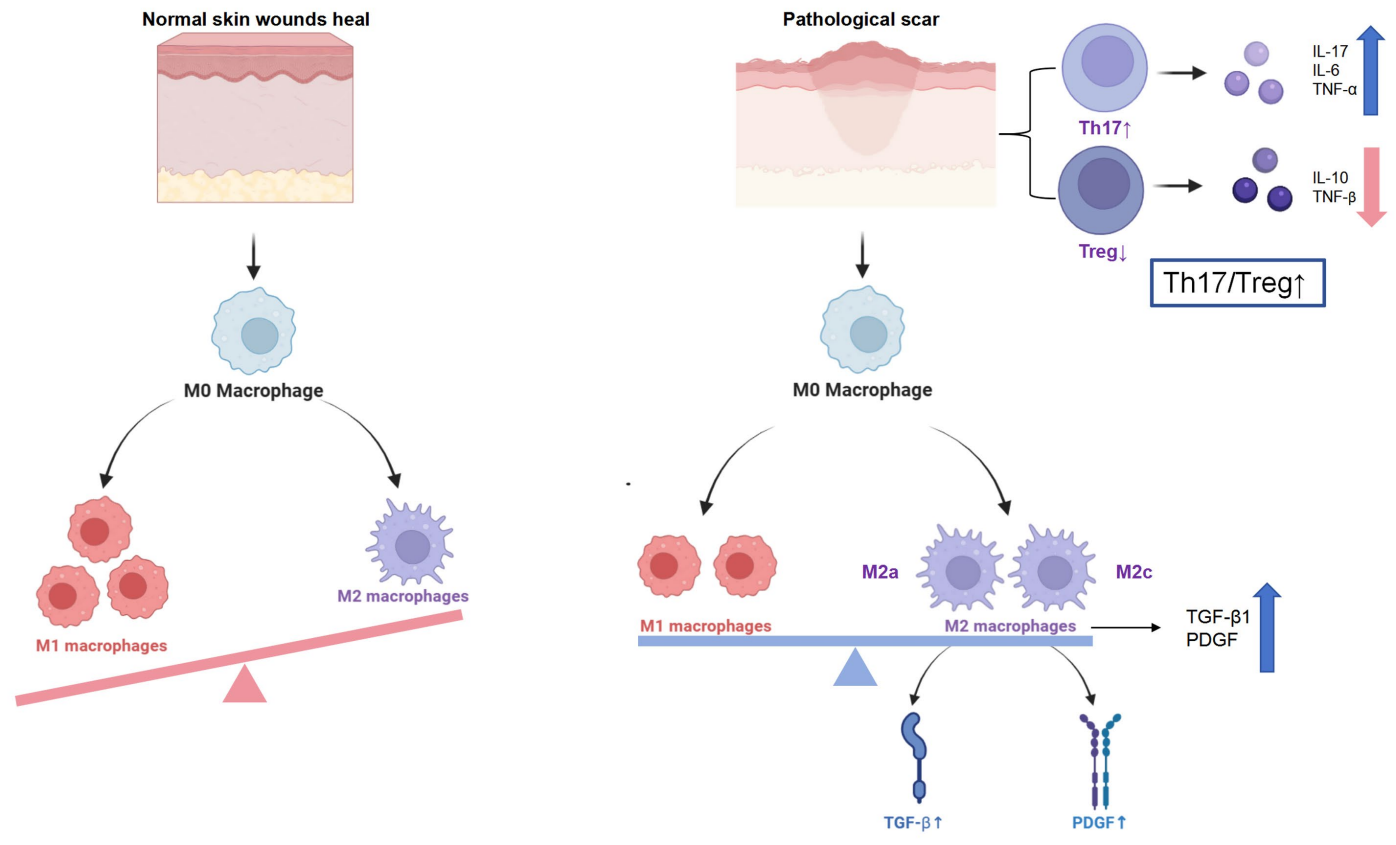
Immune dysregulation in pathological scarring. Persistent M2 skew, Th17/Treg imbalance and cytokine burst drive fibroblast activation and excessive ECM deposition in keloids and hypertrophic scars.

Emerging evidence also implicates epigenetic dysregulation within immune cells as a contributing factor to this immune imbalance. Aberrant patterns of histone acetylation and DNA methylation have been identified in macrophages and T cells isolated from keloid tissues, correlating with sustained M2 polarization and enhanced Th17 activity (Stevenson et al., 2021). These findings suggest that targeting key epigenetic regulators—such as histone deacetylases (HDACs) and DNA methyltransferases (DNMTs)—may offer a novel strategy to restore immune homeostasis and mitigate fibrotic progression (Almier et al., 2025; Figure 2).

## 3 Systemic roles of gut microbiota in fibrotic diseases

The gut microbiota, as a vast and dynamic microbial ecosystem within the human body, plays essential roles not only in nutrient digestion and metabolic homeostasis but also in regulating the development, maturation, and function of the host immune system. In recent years, increasing evidence suggests that the gut microbiome and its metabolites can modulate both local and systemic immune responses—impacting the pathophysiology of distant organs, including the skin. This emerging understanding offers new insights into the systemic contributors to pathological scarring, such as hypertrophic scars and keloids.

### 3.1 Gut microbiota composition and its direct role in fibrosis

Emerging evidence supports a critical role for both gut and skin microbiota in modulating fibrotic processes—extending beyond their secreted metabolites to include structural components and immune interactions. However, it is essential to distinguish between signals specific to the gut microbiota versus those potentially derived from the skin microbiome, particularly in understanding systemic versus local drivers of pathological scarring.

Recent multi-omics analyses have revealed distinct alterations in the gut microbiota of individuals with multiple keloids. Species such as *Oxalobacter formigenes*, *Bacteroides plebeius*, and *Parabacteroides distasonis* were significantly underrepresented in keloid-prone patients (Li et al., 2024). For instance, the loss of *B. plebeius* was associated with decreased systemic uracil levels—suggesting that gut-derived metabolic changes may influence fibroblast bioenergetics and drive fibrotic remodeling. Since uracil is not known to be synthesized by cutaneous microbes in significant amounts, these effects likely reflect a gut-specific metabolic deficit.

In contrast, localized dysbiosis within keloid tissues, especially increased colonization by pro-inflammatory bacteria such as *Staphylococcus aureus*, has been associated with elevated IL-8 signaling. This promotes fibroblast migration and collagen accumulation via the CXCR1/2 pathway (Zhang et al., 2024). Unlike gut-derived metabolites, these effects are largely restricted to the wound microenvironment, representing skin-resident microbial contributions to scar formation.

Furthermore, bacterial structural components, including lipopolysaccharides (LPS), peptidoglycan, and flagellin, are shared by both gut and skin microbes, but their systemic impact is often rooted in gut barrier dysfunction. For example, LPS—a component of Gram-negative bacterial outer membranes—is primarily absorbed into circulation when intestinal permeability is compromised, a process largely influenced by gut microbial balance and mucosal health. Once in the bloodstream, LPS activates Toll-like receptor 4 (TLR4), triggering systemic inflammation through MyD88/NF-κB signaling and inducing IL-6, TNF-*α*, and other pro-fibrotic mediators (Wenying et al., 2023).

Although LPS is not exclusive to the gut microbiota, its fibrosis-related systemic effects are most pronounced under conditions of gut-origin endotoxemia. For instance, *in vitro* exposure of hypertrophic scar fibroblasts to LPS enhances expression of collagen I/III and α-SMA (Xu et al., 2023). LPS also synergizes with TGF-*β* to augment fibrotic signaling (Yang et al., 2013), while animal studies demonstrate that systemic or oral LPS exposure aggravates scarring, reinforcing the gut’s contribution to dermal fibrosis (Xu et al., 2023). Collectively, these findings emphasize that bacterial structural components—particularly LPS—contribute to fibrosis mainly through inflammation-driven pathways and modulation of fibroblast sensitivity.

### 3.2 Microbial metabolites and their role in pathological scarring

Gut microbiota-derived metabolites have emerged as key regulators of host immune balance, epithelial barrier integrity, and fibrotic remodeling, with increasing relevance to skin disorders such as hypertrophic scars and keloids. In the context of the gut–skin axis, dysbiosis of intestinal microbiota can influence skin fibrosis through both immune modulation and translocation of microbial products, ultimately shaping fibroblast activation and collagen deposition.

#### 3.2.1 Short-chain fatty acids

Short-chain fatty acids (SCFAs)—primarily acetate, propionate, and butyrate—are generated through the anaerobic fermentation of non-digestible carbohydrates by commensal gut microbes. These carbohydrates include inulin-type fructans (ITFs), fructooligosaccharides (FOS), galactooligosaccharides (GOS), resistant starches (RS1–RS4), arabinoxylans, pectins, and *β*-glucans, each with distinct plant origins such as chicory root, grains, legumes, or citrus (Baxter et al., 2019). These substrates escape enzymatic digestion in the small intestine and are selectively metabolized in the colon by bacterial genera including Faecalibacterium, Roseburia, Eubacterium, Prevotella, and Bacteroides. For instance, *Ruminococcus bromii* and *Eubacterium rectale* specialize in degrading resistant starches to yield butyrate, while Bifidobacterium ferments GOS and FOS into acetate and lactate, which can then be cross-fed into butyrate synthesis by other microbes (Liang et al., 2023). The resulting SCFAs serve both local and systemic functions: butyrate supports colonic epithelial health by acting as a primary energy source for colonocytes and by upregulating tight junction proteins (e.g., Claudins, ZO-1, Occludin); acetate and propionate enter circulation to influence lipid metabolism, gluconeogenesis, and immune responses via G-protein coupled receptors GPR41/43 (Ribeiro et al., 2022). Importantly, butyrate also acts as a histone deacetylase (HDAC) inhibitor, thereby promoting regulatory T cell (Treg) differentiation and anti-inflammatory cytokine (IL-10) expression, while suppressing TNF-*α* and other pro-inflammatory mediators (Corrêa-Oliveira et al., 2016).

Beyond the gut, SCFAs exert systemic immunomodulatory effects by activating GPR41/43 and influencing the Th17/Treg balance. In murine models of psoriasis, butyrate restored Treg function via IL-10 and FoxP3 upregulation through HDAC inhibition (Kim, 2018). Given that pathological scarring is marked by Th17 dominance and Treg deficiency, SCFA depletion in dysbiotic states may shift the immune landscape toward fibrosis. Supporting this, metabolomic profiling of keloid tissues has revealed reduced SCFA concentrations, implying a potential role in scar pathogenesis (Yang et al., 2022).

#### 3.2.2 Tryptophan metabolites

Tryptophan metabolism by gut microbiota yields a wide range of bioactive compounds that influence host immune responses and fibrotic outcomes, particularly through activation of the aryl hydrocarbon receptor (AhR). Key microbial species, including Lactobacillus, Bifidobacterium, and Clostridium spp., convert dietary tryptophan into AhR ligands such as indole-3-propionic acid (IPA), indole-3-aldehyde (IAld), and indole-3-acetic acid (IAA), which have been shown to enhance epithelial barrier function, reduce pro-inflammatory cytokine signaling, and promote anti-fibrotic immune regulation (Bahman et al., 2024; Stec et al., 2023). These metabolites stimulate AhR in immune and epithelial cells, leading to downstream IL-22 production. IL-22 contributes to tissue repair and immune homeostasis by supporting epithelial integrity, antimicrobial defense, and regulation of Th17/Treg balance—all critical elements in wound resolution and prevention of dermal fibrosis (Mar et al., 2023). Importantly, AhR signaling also influences anti-fibrotic pathways in skin-resident immune cells. For example, IAld was shown to activate Langerhans cells to secrete IL-10 and reduce skin inflammation through AhR-dependent mechanisms, which may modulate fibroblast activation and scar formation (Liu et al., 2020). Additionally, tryptophan metabolites are capable of promoting IL-10 receptor expression and suppressing NF-κB signaling, thereby further mitigating inflammatory and fibrotic responses in epithelial tissues (Alexeev et al., 2017).

#### 3.2.3 Secondary bile acids

Secondary bile acids (SBAs) such as deoxycholic acid (DCA) and lithocholic acid (LCA) are produced by gut microbiota—especially Clostridium, Bacteroides, and Eubacterium—through 7*α*-dehydroxylation of primary bile acids (Funabashi et al., 2020). These microbial metabolites exert significant immunomodulatory and antifibrotic effects, particularly in tissues with immune-barrier functions such as the skin. LCA and DCA act as ligands for nuclear and membrane bile acid receptors, including FXR (Farnesoid X Receptor) and TGR5 (G-protein-coupled bile acid receptor 1). Through FXR activation, SBAs suppress NF-κB signaling and reduce the production of pro-inflammatory cytokines like IL-6 and TNF-α. Simultaneously, TGR5 activation skews macrophage polarization toward an anti-inflammatory M2 phenotype, promoting IL-10 production and inhibiting fibrotic signaling cascades (Mikó et al., 2018). Importantly, secondary bile acid supplementation has been shown to reverse skin inflammation in diet-induced psoriatic models by modulating the IL-17/Th17 axis and enhancing TGR5 expression in keratinocytes (Bloomstein et al., 2022). This highlights a systemic link between gut-derived bile acids and skin immune regulation. Furthermore, LCA not only reduced IL-17A expression in skin T cells but also inhibited chemokine (CCL20) expression in keratinocytes, limiting pro-inflammatory T cell infiltration (Shi et al., 2022). However, dysbiosis-induced loss of SBA-producing bacteria (e.g., Lachnospiraceae) leads to a decline in circulating SBAs and favors a pro-inflammatory, fibrosis-prone skin microenvironment (Bloomstein et al., 2022). This gut-skin axis disruption is increasingly recognized in chronic dermatoses and fibrotic skin conditions.

#### 3.2.4 Linking metabolites to clinical and genetic evidence in pathological scarring

Recent Mendelian randomization and microbiome-wide association studies provide evidence that specific microbial taxa correlate with fibrotic skin phenotypes. For instance, Melainabacteria and Negativicutes were positively associated with increased keloid risk (OR = 1.48 and 1.35, respectively), while Intestinimonas and Ruminococcus2 were protective against hypertrophic scar formation (OR = 0.71 and 0.68; Shucheng et al., 2024; Xue et al., 2024). Clinical 16S rRNA analyses further showed that individuals predisposed to abnormal scarring had lower gut microbial diversity and shifts in Firmicutes, Bacteroides, and *Escherichia coli* abundance compared to normal healers (Li et al., 2023).

In addition to microbial metabolites, compromised gut barrier function in dysbiosis may allow translocation of microbial components such as lipopolysaccharide (LPS) and CpG-rich bacterial DNA into systemic circulation. LPS activates TLR4 on dermal fibroblasts and macrophages, upregulating TGF-β1 and collagen I expression via NF-κB signaling, while CpG-DNA engages TLR9 to further induce IL-6 and TGF-β1 secretion (Li et al., 2019; Shao et al., 2022)—a mechanism known to promote fibrosis.

Animal studies underscore the immunologic consequences of these disruptions: Treg depletion increases *α*-SMA and collagen deposition in fibroblasts, while GATA-3 knockout enhances dermal fibrosis (Kalekar et al., 2019). Conversely, IL-17A—overexpressed in Th17-dominant states—can suppress Treg differentiation and exacerbate fibrotic remodeling by repressing TGF-β1 and FoxP3 expression (Dufour et al., 2020).

Taken together, these findings reveal that gut microbial composition and its metabolites—SCFAs, tryptophan derivatives, and bile acids—modulate systemic immune responses and fibroblast activation through multiple convergent mechanisms. Alterations in these metabolites, compounded by increased microbial translocation and immune imbalance, may drive susceptibility to pathological scarring. Further mechanistic and interventional studies are warranted to validate causality and explore microbiota-targeted antifibrotic strategies.

### 3.3 Mutual influence between skin and gut microbiota

The skin and gut constitute the body’s two largest epithelial-immune interfaces. Both are richly vascularized and innervated neuro-endocrine organs that constantly sample resident microbes and metabolites to calibrate local and systemic immunity. Accumulating evidence shows that dysbiosis in either niche reverberates across the other via hormonal, neural and cytokine circuits, pre-disposing individuals to chronic inflammatory dermatoses and defective wound repair (De Pessemier et al., 2021; Clarke et al., 2014).

#### 3.3.1 Gut-derived signals that restrain cutaneous fibrosis

Commensal bacteria generate short-chain fatty acids (SCFAs), secondary bile acids, GABA, serotonin, dopamine and other bio-actives that enter the circulation and reach the dermis. SCFAs engage the free-fatty-acid receptor GPR43/FFAR2, which was recently shown to stabilize the mechano-transducers YAP/TAZ through a RhoA-dependent pathway (Park et al., 2021). Direct anti-myofibroblast effects. *In vitro*, sodium butyrate (0–16 mM) down-regulates *α*-SMA and type I/III collagen transcription in human dermal fibroblasts; co-treatment with docosahexaenoic acid (DHA) potentiates this anti-fibrotic effect (Maeshige et al., 2019). Systemic immune modulation occurs as short-chain fatty acids (SCFAs) and gut-derived bile acids activate host receptors such as GPR43, TGR5, and FXR, leading to suppression of circulating pro-inflammatory cytokines including IL-6 and TNF-α. This signaling promotes macrophage polarization toward a pro-resolving M2 phenotype, thereby disrupting the TGF-β1–driven feed-forward loop that sustains fibrotic remodeling in keloids and hypertrophic scars (Trompette et al., 2022).

#### 3.3.2 Cutaneous dysbiosis and its potential feedback to the gut–immune axis

Emerging evidence suggests that local skin microbiota alterations may not only reflect fibrotic pathology but also modulate systemic immune and barrier function. Longitudinal analyses of burn scar maturation indicate that α-diversity increases over time (from 3 to 12 months), and microbial composition—particularly the abundance of Campylobacter (negatively correlated) and Cutibacterium (positively correlated)—associates with viscoelastic scar properties (Jung et al., 2025). In keloid tissues, chronic colonization by *Staphylococcus aureus*-rich biofilms has been shown to activate dermal macrophages, leading to upregulation of pro-fibrotic mediators. For example, stimulation with bacterial lipoteichoic acid enhances TGF-β1/Smad3 signaling in fibroblasts, promoting α-SMA expression and collagen deposition (Dokoshi et al., 2024). Such localized immune activation may have systemic consequences. Elevated levels of inflammatory cytokines such as IL-1β and IL-6—observed in fibrotic scar tissues—are known to disrupt intestinal tight junctions and compromise gut barrier integrity, which in turn can alter gut microbial composition (Kaminsky and Al-Sadi, 2021).

Although direct evidence for a reciprocal “skin-to-gut” regulatory loop remains limited, this bidirectional model aligns with broader observations in chronic inflammatory conditions (e.g., psoriasis, atopic dermatitis), where cutaneous inflammation correlates with altered intestinal permeability and dysbiosis. Therefore, it is plausible that chronic skin dysbiosis and biofilm-driven inflammation in pathological scarring may secondarily impact gut-immune homeostasis, reinforcing fibrotic signaling through systemic pathways. This hypothesis merits further investigation in mechanistic and longitudinal human studies.

#### 3.3.3 Conceptual synthesis for pathological scars

Pathological scar formation can be conceptualized as a self-propagating three-stage cascade. First, gut dysbiosis diminishes systemic pools of short-chain fatty acids and beneficial bile acids, weakening GPR43-mediated inhibition of YAP/TAZ in dermal fibroblasts and fostering persistent low-grade inflammation. Second, when skin injury occurs against this backdrop, the simultaneously altered cutaneous microbiota release enduring danger signals—such as unmethylated CpG DNA and lipoteichoic acid—that robustly activate dermal macrophages and resident fibroblasts, amplifying local cytokine circuits. Third, the resultant surge of TGF-*β*1, compounded by sustained mechanical tension across the wound bed, locks fibroblasts into a contractile myofibroblast phenotype; the cytokines generated during this phase further compromise gut-barrier integrity and restructure intestinal microbial communities, thereby aggravating the original dysbiosis and perpetuating the gut–skin–fibrosis loop.

Collectively, these multi-organ interactions provide a mechanistic basis for the clinical observation that patients with gut dysbiosis (e.g., high-fat diet, prolonged antibiotics) tend to develop more exuberant keloids or hypertrophic scars, and they rationalize emerging adjunctive therapies such as probiotics, prebiotics or SCFA-boosting diets alongside intralesional steroids or laser therapy (Table 1).

**Table 1:** Microbe-metabolite-fibrosis table.

Key microbial strain	Metabolite produced	Mechanistic impact on pathological scarring
*Faecalibacterium*, *Roseburia*, *Eubacterium*, *Bacteroides*, *Prevotella*	Short-chain fatty acids (SCFAs: butyrate, acetate, propionate)	HDAC inhibition; promotes Treg and IL-10; suppresses IL-6, TNF-α; inhibits YAP/TAZ in fibroblasts; reduces α-SMA, COL1/3
*Ruminococcus bromii*, *Eubacterium rectale*	Butyrate from resistant starch fermentation	Downregulates myofibroblast markers; enhances epithelial integrity
*Bifidobacterium* spp.	Acetate, lactate (SCFA precursors)	Supports butyrate synthesis by cross-feeding; modulates immune response
*Lactobacillus*, *Clostridium*, *Bifidobacterium*	Tryptophan metabolites (IPA, IAld, IAA)	Activate AhR signaling → IL-22, IL-10 ↑, NF-κB ↓; enhance epithelial barrier; regulate Th17/Treg balance
*Clostridium*, *Bacteroides*, *Eubacterium*	Secondary bile acids (DCA, LCA)	FXR/TGR5 activation → IL-6/TNF-α ↓, IL-10 ↑; M2 macrophage polarization; inhibit IL-17A, TGF-β1, CCL20
	LPS (mainly from Gram-negative bacteria)	Activates TLR4/MyD88/NF-κB → IL-6, TNF-α ↑; sensitizes fibroblasts to TGF-β1; promotes collagen deposition
	CpG bacterial DNA	Activates TLR9; induces IL-6, TGF-β1; promotes fibroblast proliferation and ECM accumulation
*Staphylococcus aureus* (skin microbiota)	Lipoteichoic acid	Enhances Smad3 phosphorylation in fibroblasts → increases α-SMA, collagen I/III
	Decreased *B. plebeius* (gut)	Uracil deficiency → impaired fibroblast metabolism → promotes fibrotic remodeling
*Lachnospiraceae* (reduced in dysbiosis)	Secondary bile acids (↓ in dysbiosis)	SBA reduction → IL-17A ↑, TGR5 ↓ → favors pro-inflammatory, fibrosis-prone skin environment

Summary of key microbial strains, their metabolites, and the associated mechanisms influencing pathological scarring. Effects include immune modulation, fibroblast regulation, and extracellular matrix remodeling.

## 4 Microbial-epigenetic crosstalk in fibrotic skin remodeling: roles of SCFAs, bile acids and non-coding RNAs

### 4.1 Epigenetic regulators of fibrotic signaling

Gut microbiota-derived signals profoundly influence fibrotic remodeling through modulation of canonical signaling pathways such as TGF-*β*/Smad, Wnt/β-catenin, and NF-κB—all of which are overactive in pathological scarring. For example, lipopolysaccharide (LPS)—a structural component of Gram-negative bacteria—can translocate systemically in cases of gut barrier dysfunction and activate Toll-like receptor 4 (TLR4) on dermal fibroblasts. This stimulates the MyD88/NF-κB axis, resulting in the upregulation of TGF-*β*1, collagen I, and *α*-smooth muscle actin (α-SMA), promoting fibroblast proliferation and excessive ECM deposition (Li et al., 2019).

Conversely, short-chain fatty acids (SCFAs)—especially butyrate and propionate—exert antifibrotic effects by acting as histone deacetylase (HDAC) inhibitors. These SCFAs promote histone acetylation, suppressing transcription of profibrotic genes such as COL1A1 and ACTA2 in dermal fibroblasts. *In vitro* studies have shown that butyrate can induce fibroblast quiescence or apoptosis and attenuate collagen synthesis (Maeshige et al., 2019). Interestingly, SCFA levels are significantly decreased in keloid tissues compared to adjacent normal skin, highlighting a potential link between microbial metabolic loss and scar overgrowth (Shucheng et al., 2024).

Secondary bile acids also play a role in regulating fibrotic pathways. Deoxycholic acid (DCA) and lithocholic acid (LCA) activate FXR and TGR5 receptors, which suppress NF-κB and NLRP3 inflammasome signaling. At pharmacological levels, DCA can inhibit fibroblast contractility and induce apoptosis, pointing to a potential antifibrotic mechanism (Ramaesh et al., 1998). Together, these epigenetic inputs shape the activation threshold of fibroblasts, balancing between repair and pathological fibrosis.

### 4.2 Non-coding RNA crosstalk and microbial modulation

Beyond chromatin-level regulation, gut-derived metabolites influence non-coding RNA networks that fine-tune fibrotic gene expression in dermal fibroblasts. Among microRNAs, miR-21 is one of the most extensively studied in keloid and hypertrophic scarring. It is upregulated in keloid tissues and directly targets Smad7, an inhibitory Smad protein, thereby reinforcing TGF-β/Smad2/3 signaling (Li et al., 2021). Notably, miR-21 promotes fibrosis by targeting negative regulators of TGF-β signaling and enhancing extracellular matrix (ECM) deposition. Epigenetic studies have shown that miR-21 expression can be induced by environmental and metabolic factors, and its promoter is associated with active chromatin marks such as H3K4me3 and H3K27ac (Ji et al., 2024). Since SCFAs are known to affect histone acetylation, their presence or depletion may indirectly influence miR-21 transcription and thus fibrotic outcomes.

MiR-199a-5p is another key player in scar fibrogenesis. It is overexpressed in hypertrophic scars and targets caveolin-2 (CAV2), a suppressor of fibroblast proliferation and collagen deposition. Inhibition of miR-199a-5p *in vitro* reduces expression of COL1A1 and *α*-SMA in scar-derived fibroblasts (Yang et al., 2020). These miRNAs act as post-transcriptional effectors of upstream profibrotic signaling and could serve as promising therapeutic entry points.

Importantly, gut dysbiosis and metabolite imbalance may shape miRNA profiles through epigenetic remodeling. Although data in human scar tissue remain sparse, studies in other fibrotic and inflammatory models have demonstrated that butyrate, a microbial-derived short-chain fatty acid (SCFA), can influence miRNA expression profiles through histone deacetylase (HDAC) inhibition. For example, in an allergic asthma mouse model, intranasal sodium butyrate reduced airway fibrosis and inflammation, partly by modulating HDAC1 activity and suppressing NF-κB signaling—pathways that also intersect with pro-fibrotic miRNA regulation (Islam et al., 2022). Moreover, in colorectal models, butyrate exposure significantly altered the expression of miRNAs, including miR-21, miR-143, and miR-145, consistent with a role for HDAC-regulated epigenetic control of fibrosis-associated genes (Mohammadi et al., 2024). Another study using high-throughput miRNA screening confirmed that butyrate synergizes with specific miRNAs to regulate cell proliferation and matrix production, implicating HDAC inhibition in broader transcriptomic remodeling (Ali et al., 2022).

**Figure 3:**
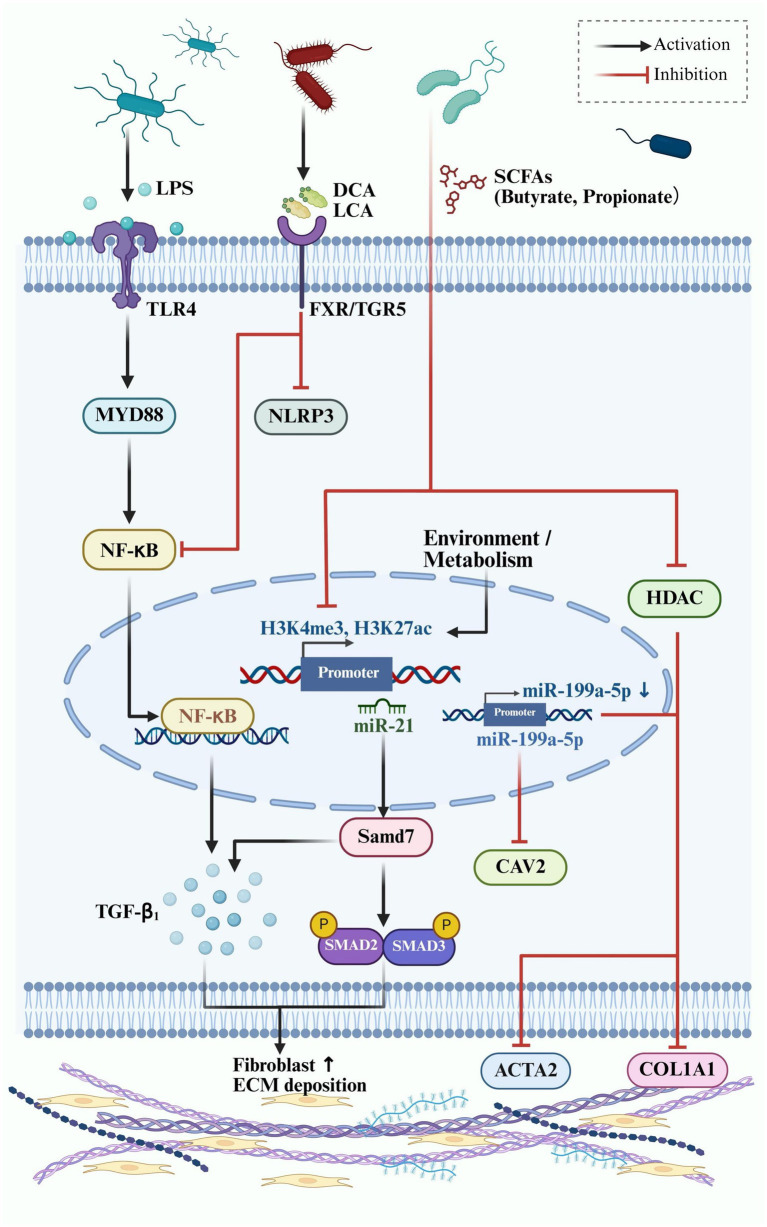
Epigenetic and microbial regulation of fibrotic signaling in dermal fibroblasts. This schematic illustrates the interaction between gut microbial metabolites and host fibrotic signaling pathways in skin fibroblasts, emphasizing epigenetic regulation.

In summary, the interplay between microbial metabolites and non-coding RNA networks establishes a molecular interface between the gut and fibrotic skin. Targeting this axis may offer dual leverage: modulating the gut microbiome to control both systemic immunity and fibroblast-specific gene expression (Figure 3).

## 5 Harnessing the gut–skin axis: emerging microbiota-based strategies for pathological scar management

### 5.1 Probiotic and prebiotic interventions

Probiotics and prebiotics are key modulators of the gut–skin axis and show considerable promise in mitigating pathological scar formation. Certain lactic acid bacteria strains—including *Lactobacillus acidophilus*, *L. casei*, and *L. rhamnosus*—have been shown to influence dermal fibroblast behavior via systemic immunomodulatory and metabolic pathways. For instance, a Lactobacillus-based oral preparation (BioK®) suppressed TGF-*β*1/Smad signaling via lactic acid production, reducing myofibroblast transdifferentiation and collagen accumulation in keloid models (Zhang et al., 2025). Similarly, extracellular vesicles from L. druckerii significantly downregulated COL1A1, COL3A1, and *α*-SMA expression in scar fibroblasts, improving scar phenotype in mice (Han et al., 2023).

Prebiotics such as inulin and fructooligosaccharides (FOS), when taken orally, enrich butyrate-producing taxa (e.g., Faecalibacterium, Roseburia) in the gut, thereby increasing the availability of short-chain fatty acids (SCFAs). These SCFAs systemically modulate key fibrotic pathways—including TGF-β/Smad and NF-κB—through GPR43/109A activation and histone deacetylase inhibition. *In vitro* studies by Maeshige et al. found that butyrate suppressed α-SMA and collagen III in human dermal fibroblasts, with synergistic effects observed when combined with omega-3 fatty acids (Maeshige et al., 2019). Probiotic lysates such as Vivomixx® (administered orally) have also shown antifibrotic activity: in human intestinal fibroblasts (CCD-18Co), the soluble fraction of Vivomixx® significantly reduced the phosphorylation of Smad2/3 and decreased collagen-I and α-SMA expression in response to TGF-β1 (Lombardi et al., 2021).

### 5.2 Dietary interventions

Dietary interventions represent another oral strategy that shapes the gut microbiota and its metabolite output, thereby influencing systemic inflammation and fibrotic signaling. Diets rich in fiber, omega-3 polyunsaturated fatty acids (PUFAs), and polyphenols enhance the production of SCFAs in the colon, strengthen epithelial barrier function, and reduce pro-inflammatory cytokines (e.g., IL-6, TNF-α). Maeshige et al. reported that an inulin-rich oral diet increased colonic butyrate levels and histone acetylation in fibroblasts, leading to suppression of fibrotic markers such as collagen and α-SMA (Maeshige et al., 2019).

Omega-3 PUFAs synergize with SCFAs to inhibit fibroblast activation by modulating lipid mediator pathways. Polyphenols like catechins and quercetin, when ingested, promote beneficial gut bacteria (e.g., Bifidobacterium), indirectly reducing IL-1*β* and TGF-β1 expression. Notably, combining dietary fiber with omega-3 PUFAs has been shown to restore mucosal barrier integrity and exert long-term antifibrotic effects (Centonze et al., 2025). These findings suggest dietary strategies may offer accessible, non-invasive options for scar prevention and control (Bavaro et al., 2024).

### 5.3 Fecal microbiota transplantation

Although direct evidence for the use of fecal microbiota transplantation (FMT) in pathological scar management is still lacking, studies in systemic fibrotic and chronic wound models suggest that modulating gut microbial communities may yield anti-fibrotic benefits. FMT is, by definition, administered via gastrointestinal routes (oral capsule or enema), thus directly reshaping the gut microbiota. In a recent study, Peng et al. demonstrated that FMT accelerated wound healing in diabetic mice by restoring beneficial genera such as Bacteroides and Lactobacillus, which was associated with activation of the IL-17A-mTOR-HIF-1α signaling cascade. This intervention led to improved fibroblast remodeling, enhanced collagen alignment, and faster re-epithelialization (Peng et al., 2025). While these findings highlight the potential of microbiota modulation to influence wound microenvironments, the translation of such benefits to fibrotic scar modulation remains hypothetical. Nevertheless, given the shared features of chronic wounds and fibrotic tissues—such as persistent inflammation, dysregulated fibroblast activity, and altered immune-metabolic profiles—FMT may warrant investigation as a microbiome-targeted strategy to influence fibrotic progression, albeit with cautious interpretation.

### 5.4 Metabolite-targeted drug development

Targeting gut microbiota-derived metabolites and their corresponding host receptors presents a promising precision-based strategy for antifibrotic therapy. Receptors such as the farnesoid X receptor (FXR) and TGR5, which detect secondary bile acids, have been shown to attenuate fibrosis by downregulating profibrotic mediators like TGF-β and CTGF, reducing collagen accumulation and inflammation in models of liver and skin disease (Kim, 2018; Zhao et al., 2023). The aryl hydrocarbon receptor (AhR), a key sensor of microbial tryptophan metabolites like quinolinic acid, can suppress the NLRP3 inflammasome and modulate immune responses, including Treg/Th17 balance. In psoriasis models, AhR activation by microbial metabolites alleviated inflammation and fibroblast activation via NLRP3 inhibition (Qiao et al., 2022). Short-chain fatty acids, especially butyrate, have demonstrated antifibrotic and anti-inflammatory properties through histone deacetylase inhibition and modulation of GPR43-mediated NLRP3 deactivation. SCFA supplementation in animal models reduced fibrotic remodeling, collagen deposition, and local inflammation (Zuo et al., 2022; Fujiwara et al., 2018). Finally, targeting the NLRP3 inflammasome with inhibitors such as MCC950 has shown significant efficacy in preventing fibroblast-to-myofibroblast transition and collagen overproduction, thereby directly intervening in scar pathophysiology (Artlett, 2012). Together, these strategies demonstrate the therapeutic potential of microbiota-derived metabolites in modulating fibrosis-related pathways, offering mechanistic depth and translational relevance for scar management.

## 6 Multi-omics and microbiome-based interventions in pathological scar management

Building on the microbiome-targeted strategies outlined above, current multi-omics approaches are being harnessed to discover novel biomarkers and metabolite-driven therapies for pathological scars. By integrating data from metagenomics, metabolomics, transcriptomics, and proteomics, researchers can obtain a comprehensive, systems-level understanding of host–microbiome interactions in fibrosis (Yang et al., 2025). This holistic view enables the identification of key microbial species, metabolites, and pathways that drive scar formation, unveiling targets for therapeutic intervention. For instance, an integrative multi-omics study in keloid patients revealed that loss of a beneficial gut commensal (*Bacteroides plebeius*) may lower levels of a metabolite (uracil) crucial for fibroblast metabolism, potentially promoting pathological scarring. These findings highlight new opportunities for therapy, as restoring such microbial functions or metabolite levels could help rebalance wound healing (Li et al., 2024). Moreover, multi-omics analyses facilitate the strategic design of microbiome-targeted interventions—including optimized probiotic consortia, precision dietary regimens, or metabolite-derived therapeutics—to translationally address scar formation (Kwoji et al., 2023). Notably, a recent Mendelian randomization and microbiome study showed that specific dietary habits (e.g., high sugar intake or certain food preferences) and certain gut bacteria independently influence hypertrophic scar risk, underscoring the potential of both diet modification and microbial modulation as preventive strategies (Liu et al., 2025).

Advances in multi-omics technologies are fundamentally reshaping our understanding of scar pathophysiology by enabling the integrative analysis of microbial, immune, and fibrotic signaling networks. In keloid patients, combined stool metagenomics, plasma metabolomics, and single-cell RNA sequencing (scRNA-seq) have uncovered associations between gut dysbiosis, altered fibroblast transcriptomic states, and aberrant collagen remodeling (Li et al., 2024). These analyses revealed enrichment of fibrogenic fibroblast subtypes and immune compartments skewed toward Th17 polarization and pro-fibrotic macrophage activation (Zhao et al., 2025). Recent translational advances leverage microbiome-based therapeutics for fibrotic modulation. Engineered probiotics, such as *Lactococcus lactis* strains engineered to secrete IL-4, macrophage colony-stimulating factor (M-CSF), and fibroblast growth factor 2 (FGF-2), are undergoing early-phase clinical evaluation for chronic wound healing (e.g., AuP1602-C; Freedman et al., 2023). Similarly, a topically applied CXCL12-producing Limosilactobacillus reuteri strain (ILP100-Topical) has demonstrated human safety and pro-healing effects in cutaneous injury models (Öhnstedt et al., 2023). Moreover, postbiotic derivatives and bacterial extracellular vesicles are being tested for regenerative skin applications with promising immunoregulatory and tissue-repair properties (Prajapati et al., 2025). In parallel, genetic interventions such as CRISPR–Cas9 editing, siRNA delivery, and targeted antisense oligonucleotides have shown preclinical efficacy in reducing TGF-β1, COX-2, and COL1A1 expression, thereby attenuating fibrosis in both murine and ex vivo scar models (Daliri et al., 2025; Fu et al., 2024). At the spatial level, high-resolution multi-omics technologies are emerging as powerful tools to decipher scar biology. Spatial transcriptomics platforms, such as Slide-seq2 and spatial host–microbe sequencing (SHM-seq), enable *in situ* mapping of gene expression, microbial localization, and immune interactions at near-cellular resolution. While SHM-seq has yet to be applied directly to skin fibrosis, its ability to resolve microbial signals within specific host cell niches holds promise for future scar microenvironment profiling (Lötstedt et al., 2024). These integrative, multi-modal approaches underscore a growing translational frontier where host–microbe–fibrosis networks can be mapped, targeted, and therapeutically rewired.

## 7 Conclusion

Pathological scarring, including hypertrophic scars and keloids, is increasingly recognized not merely as a localized dermal abnormality but as a systemic fibroinflammatory condition shaped by the gut–skin axis. Emerging evidence highlights the critical role of gut microbiota-derived metabolites—such as short-chain fatty acids, secondary bile acids, and tryptophan derivatives—in modulating immune polarization, fibroblast activation, and extracellular matrix remodeling, with additional regulation through epigenetic mechanisms including non-coding RNAs and histone modifications. Advances in multi-omics technologies, including metagenomics, metabolomics, and single-cell transcriptomics, are reshaping our understanding of scar pathogenesis by uncovering intricate host–microbiota interactions and identifying potential microbial or metabolic biomarkers. At the therapeutic level, microbiota-targeted interventions—ranging from probiotics and prebiotics to postbiotics, metabolite-based drugs, and even fecal microbiota transplantation—offer promising strategies to modulate fibrotic progression and improve treatment outcomes. Looking forward, future research should aim to define causal links, stratify patient-specific microbial signatures, and develop microbiome-informed, precision-based therapies. Together, these insights not only broaden our conceptual framework of pathological scarring but also open new avenues for systemic, mechanism-driven treatment approaches beyond traditional local interventions.
